# Teachers: the forgotten health workforce

**DOI:** 10.1177/01410768221085692

**Published:** 2022-03-10

**Authors:** Chloe Lowry, Rosie Leonard-Kane, Ben Gibbs, Lisa-Maria Muller, Alison Peacock, Anant Jani

**Affiliations:** 1 Institute of Education, University College London, London WC1H 0AL, UK; 2 UCD Innovation Academy, University College Dublin, Dublin D04 N2E5, Ireland; 3 RestartEd Ltd, Cambridge CB6 1DT, UK; 4 Chartered College of Teaching, London WC1N 1AZ, UK; 5 Heidelberg Institute of Global Health, University of Heidelberg, Heidelberg 672 69120, Germany

The mental health of children and young people in England, and the services designed to support them, are in a dire state. Rates of mental illness among children have increased by 50% in just three years. Provision is nowhere near sufficient to meet need, and, in an appalling failure of ‘parity of esteem' with physical health, the NHS currently aims to treat only 1/3rd of children with mental health conditions.^1^

The level of investment in children's mental health services, access rates and wait times varies wildly across the country.^2^ However, almost every child in the country has direct access to a trusted professional with a duty to promote their welfare: a teacher. Indeed the Mental Health of Children and Young People in England (MHCYP) survey shows that teachers are the most common source of mental health support for children (Figure 1).^3^

**Figure 1.**
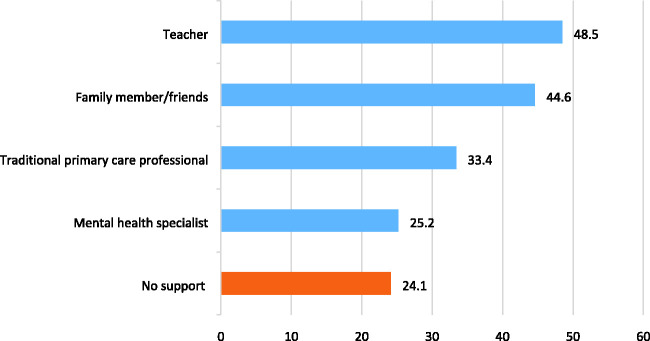
Types of support contacted for mental health in the past year for 5–19-year-olds with a disorder (adapted from NHS Digital^3^). Note: As more than one type of support can be sought, percentages do not sum to 100.

Schools are ideal locations for children to access help, as they can do so directly and independently. Yet schools have experienced real-terms cuts in funding.^4^ Teachers are not adequately trained to promote mental health or respond to problems.^4,5^ Like those on the frontline of healthcare, disproportionate numbers of teachers experience poor mental health themselves.^6^

This commentary highlights the role teachers play in children's health and argues that more funding should be allocated to support and equip this forgotten health workforce.

## Teachers' dual role in children's health

Teachers play both public health and primary care roles, promoting societal health and acting as the first point of contact when problems arise.

### Teachers as public health professionals

Longitudinal research shows that teachers significantly impact the long-term physical and mental health of their pupils. Children's sense of being valued by their teachers and peers, known as school connectedness, impacts rates of substance misuse, early sexual initiation, violence, injury, emotional distress, disordered eating and suicide. School connectedness is particularly important for vulnerable children as it can compensate for low connectedness in other areas of life.^6^

Research indicates that the pathway by which school connectedness impacts long-term health is the pupil–teacher relationship. An intriguing study separated out the health impact of positive pupil–pupil versus pupil–teacher relationships, using sibling fixed-effect modelling to comprehensively control for family background. It revealed that the associations between pupil–pupil relationships and health in adulthood were mostly driven by family-level factors. However, the associations between pupil–teacher relationships and adult health were robust, with the quality of the relationship significantly predicting general health, risk of cardiovascular disease, depression scores, smoking and binge drinking in adulthood.^7^

Ground-breaking research by the Centre for Economic Performance (CEP) has shown that the influence of individual teachers on pupils' mental health is as significant as their influence on academic test scores. Analysis of rich longitudinal data from 10,000 British primary school pupils found that their emotional symptoms, conduct problems, hyperactivity/inattention, peer relationships and prosocial behaviour were significantly impacted by their teachers. Whereas a teacher's influence on pupils' test scores faded rapidly after pupils graduated their class, their impact on social and emotional learning (SEL) persisted. Intriguingly, the ability to promote SEL was only weakly correlated with the ability to improve test results, indicating that different skillsets and training are required. Teachers' ability to promote SEL has long-run implications: replacing a teacher in the bottom 10% of SEL-promotion ability with a teacher of mean ability would generate an estimated lifetime earnings gain of £25,000 per child taught.^8^

### Teachers as primary care professionals

Alongside promoting long-term health, teachers provide primary care to children and young people. Together with professionals like general practitioners and social workers, teachers constitute ‘tier 1' of the Child and Adolescent Mental Health Service (CAMHS) and are considered able to ‘offer general advice and treatment for less severe problems … contribute towards mental health promotion, identify problems early in the child or young person's development, and refer to more specialist services.’^9^

In practice, tier 1 professionals end up providing crucial support for children across the spectrum of need, because it is difficult to access specialist services: only a quarter of children with a mental disorder in England receive specialist support.^1^ In a national scoping study of mental health provision in English schools, staff with no specialist mental health training were reported to be the main people in school providing help to pupils.^10^

The 2017 MHCYP survey, a nationally representative sample of 5–19-year-olds in England, revealed that teachers were the primary access point for children's mental health support, being contacted for support for half of children with a disorder (Figure 1). It was even more common to contact a teacher than to seek support informally from family or friends. Worryingly, a quarter sought no support at all.

Moreover, teachers were overwhelmingly more likely than other professionals to be contacted for support for children *without* a mental disorder.^3^ Many of these will be children with ‘pre-diagnosable conditions' (estimated at 1.2 million in England), who have symptoms of a disorder and would benefit from support, but do not reach a diagnostic threshold and would not be eligible for NHS treatment.^2^ This highlights that teachers are not only the first port of call when concerns arise, but for many the only port of call, making their response key to determining future outcomes.

After educational support services, teachers were the professional service most likely to be considered helpful when contacted (72%), and it was uncommon for teachers to be considered unhelpful (10%) – much less than traditional primary care professionals (17%).^3^ Unlike other tier 1 professionals, education professionals see children's emotions and behaviours day-to-day and have an ongoing relationship with them, making them ideally placed to assess and respond to concerns.

The most recent findings are even more stark: in 2020–2021, education services were contacted for mental health support for those with a probable disorder at approaching double the rate of health services.^11^

Teachers' roles go even further. A recent report found that during pandemic-related school closures, families turned to teachers for support with issues from financial and housing worries to domestic violence and bereavement. It is evident that teachers provide society with vital services beyond education.^12^

It is therefore both astonishing and alarming that teachers in England are not adequately trained for these roles.

## The untrained professionals

A survey of initial teacher training (ITT) providers in England found that coverage of health and wellbeing was notably variable, and that the level of training depended on the priorities of the individual schools where trainee teachers completed placements.^5^ In an international survey of teachers, the majority of UK respondents reported that they had never been trained in SEL.^13^ The national scoping survey found that mental health training for staff was uncommon, with most schools providing it ‘not at all' or ‘a little'.^10^ The government currently funds mental health awareness training for only one teacher per school.^2^

The result is that, despite being tier 1 CAMHS professionals, just 40% of classroom teachers in England reported feeling equipped to teach children in their class who have mental health needs, and only 32% knew how to help pupils access specialist mental health support outside school.^14^ A survey of UK teachers during the COVID-19 pandemic found that over half were not confident in supporting grieving or traumatised children. Mental health training was the most sought-after type of training among respondents.^12^

Despite this background of unprecedented need, inadequate training and a workforce eager to learn, training in promoting children's healthy development was omitted in the final stages of recent ITT reforms in England. The 2015 governmental review into ITT provision recommended the development of a core curriculum for all providers. It covered children's emotional development and mental health, and would have trained teachers to recognise atypical development and respond appropriately.^15^ This curriculum was accepted by the government; however, the finalised version omits this essential content entirely, in favour of a narrow focus on improving academic attainment through promoting high-quality teaching.^15,16^

## Towards a healthier education system

Wellbeing and academic outcomes are not competing priorities. The CEP research found that children's long-run outcomes were improved by a combination of teachers' impact on test scores and on SEL.^8^ Young people with good social and emotional development achieve better GCSE results.^17^ Children's lives are not a zero-sum game.

Pupils are not the only ones suffering under the current system. Teachers report significantly higher levels of work-related stress and poorer mental health than other occupations.^6^ A large-scale survey of UK education professionals found that 10% reported feeling suicidal as a result of work, and 50% had considered leaving due to pressures on health and wellbeing, with the volume of workload cited as the main pressure.^18^ Much of this work may be unnecessary: preliminary research in England found that teachers' workload can be reduced, improving teacher wellbeing while maintaining or improving pupils' academic outcomes.^19^

This has consequences beyond teachers' long-term health and staff retention. Research shows that children's mental health and wellbeing is associated with that of their teachers, unsurprising considering the significance of the pupil–teacher relationship.^6^ In the CEP study, teachers' emotional health was significantly related to their ability to improve both children's academic results and SEL.^8^ These relationships are likely to be bi-directional, with poor teacher wellbeing negatively impacting pupil wellbeing and attainment, and vice versa.

All of this indicates that the education system does not currently promote the long-term health and wellbeing of those within it, and may even be harmful. We offer three recommendations to create a healthier education system, turning vicious cycles of poor pupil and teacher wellbeing into virtuous circles that enhance children's long-term physical health, mental health, educational and economic outcomes.

### Train the workforce

Teachers are not health professionals, but they should be experts in promoting healthy child development and confident in providing basic mental health support. We recommend that comprehensive training in child development, health and wellbeing is integrated into ITT and the Early Career Framework, and provided free of charge to all current teachers.

Teachers' roles vary so different training pathways tailored to different specialised roles should be developed, e.g. for form teachers, pastoral managers, health education teachers and school leaders.

Learning from the limitations of a previous training initiative ‘MindEd', an online portal on child mental health for all UK adults, all training should be accredited, tailored to the school context, and include in-person elements.^20^

### Measure what matters

Academic attainment is far from the only valuable output of the education system. All the modifiable factors relevant to children's long-term success should be measured regularly. We recommend collecting anonymous school-level data on pupil health, wellbeing, and SEL, pupil–teacher relationships, and teacher wellbeing and workload. This would equip teachers to understand the needs of their pupils, and schools to understand the needs of their teachers, and respond accordingly.

As explored elsewhere in this *JRSM* series, these data could underpin feedback loops on both local and national scales to inform future practice and teacher training, creating a ‘Learning Education System' that makes schools ever-healthier places to teach and learn.^6,17^

### Create child-centred services

Essential services should be made available to children where they are: school. Teachers are the ideal first access point in child-centred services. Yet for the sake of both children and teachers, teachers must not be children's only source of professional help.

The government commitment to create school-based ‘Mental Health Support Teams' in 20% of areas is a welcome development.^2^ We recommend going further, and investing in schools as centres of expertise on children's health and wellbeing, and as hubs for all children's services, from social workers to social prescribing link workers.

## Conclusion

Children's burgeoning health needs are not currently being met by the health sector. Schools and teachers provide vital support, but they are buckling under the strain of the demands placed on them. To the extent that they perform public health and primary care roles, they should receive funding to support them to deliver these functions. Furthermore, it would be reasonable for this funding to come from the health sector, as highlighted in another manuscript in this series, given the essential role schools and teachers play in supporting children's health and wellbeing.^21^ The scale of investment in them must match the scale of the task they undertake: educating the nation's children, promoting their healthy development, and providing them with frontline health and wellbeing services.

## Declarations

### Guarantor

AJ.
